# Increased Left Inferior Temporal Gyrus Was Found in Both Low Function Autism and High Function Autism

**DOI:** 10.3389/fpsyt.2018.00542

**Published:** 2018-10-30

**Authors:** Jia Cai, Xiao Hu, Kuifang Guo, Pingyuan Yang, Mingjing Situ, Yi Huang

**Affiliations:** ^1^Mental Health Center, West China Hospital of Sichuan University, Chengdu, China; ^2^Psychiatric Laboratory, State Key Laboratory of Biotherapy, West China Hospital, Sichuan University, Chengdu, China; ^3^Brain Research Center, West China Hospital of Sichuan University, Chengdu, China

**Keywords:** low functioning autism, high functioning autism, inferior temporal gyrus, VBM, structural MRI

## Abstract

Previous neuroimaging studies of autism spectrum disorder (ASD) have focused on subjects with IQ > 70 or ASD without considering IQ levels. It remains unclear whether differences in brain anatomy in this population are associated with variations in clinical phenotype. In this study, 19 children with low functioning autism (LFA) and 19 children with high functioning autism (HFA) were compared with 27 healthy controls (HC). We found increased gray matter volume (GMV) in the left inferior temporal gyrus in subjects with both HFA and LFA and increased GMV of left middle temporal gyrus BA21 was found only in the LFA group. A significant negative correlation was found between the left inferior temporal gyrus (LITG) and the score of repetitive behavior in the HFA group.

## Introduction

Autism spectrum disorder (ASD) is a neurodevelopmental disorder that presents with social communication deficits and restricted, repetitive patterns of behavior, interests, and activities (1). ASD is reported to affect 1 in 59 individuals according to the last CDC update of autism's estimated prevalence (2). Among the patients, more than 80% are male and about 70–80% have intelligence disabilities (ID) (3, 4). The symptoms of ASD negatively impact the patients' social outcomes and consequently they become a tremendous burden on both the family and community (5).

According to the 10th revision of the International Statistical Classification of Diseases (ICD-10) by the World Health Organization (WHO) in 2016, there is clinical heterogeneity within this disorder, including cognitive functioning, behaviors, and sensory-motor functioning. Subjects with an IQ < 70 are typically defined in the literature as having low functioning autism (LFA) and those with an IQ ≥ 70 are defined as having high functioning autism (HFA). Those who have normal or even superior intellectual abilities and have no history of developmental language delay are classified as having Asperger syndrome (6, 7). Because of its heterogeneity and frequent comorbidities (epilepsy, intelligence disability, or other mental disorders), it is difficult to elucidate the underlying biological causes of ASD (8, 9). It has been hypothesized that environmental, immunological, and genetic factors all were involved in its etiology (10, 11). Exploring the biological markers for the heterogeneous phenotypes of ASD, using techniques such as magnetic resonance imaging (MRI) of the brain, can help us to better understand the underlying disease mechanisms and improve patient outcomes by targeted interventions.

Several Voxel-based morphometry (VBM) studies (using an MRI technique to investigate focal differences in brain anatomy) have demonstrated abnormalities in the right fusiform gyrus, right temporal-occipital lobe, left parietal lobe, left middle temporal gyrus, and superior temporal gyrus in patients with ASD, but the conclusions have been inconsistent (12–15). Furthermore, abnormalities were also found in subcortical areas, including increased volume in the caudate nucleus and hippocampus, and decreased volume in the cerebellum and basal ganglia (16–18). Evidence from HFA patients has also shown correlations between clinical symptoms, cognitive functioning, and abnormal regional brain volume. For example, caudate nucleus, parietal lobe, and temporal lobe activity are involved in the repetitive behaviors of ASD patients (19), and the cerebellum is associated with the communication deficits and repetitive behaviors (20). A recent study found that increased gray matter volume (GMV) in the left temporal gyrus was correlated with social interactions and frontal lobe changes were associated with more severe repetitive symptoms in adults with ASD (21). Three studies showed that ASD patients may have an abnormal structure of the “social brain” that may be associated with development of social communication disorder (22–24). The relationship between the autistic symptoms, cognitive functioning and abnormal brain volumes was not clear and most of the studies examining neuroimaging features and manifest behaviors have been conducted in HFA subjects, making it unclear as to whether the results can be generalized to LFA subjects who account for nearly 80% of the ASD population.

Previously, we found four VBM studies which took the individuals' cognitive function into account by separating the whole sample into LFA and HFA. Toal et al. (25) found that adults with ASD had significant reductions in the gray-matter volume of the medial temporal, fusiform, and cerebellar regions that varied with clinical phenotype. Adult patients with autism also demonstrated an increase in gray matter in the frontal and temporal lobes (25). An additional three studies were focused on children with ASD. Riva et al. (18) reported that decreased GMV regions were found in the basal forebrain, nucleus accumbens, and cerebellar hemispheres in the LFA group. Furthermore, the reduction of GMV in the Vermis and CRUS-II was associated with social and interaction deficits in LFA patients (26). Another recent study found increased GMV in the left superior temporal gyrus and left postcentral gyrus in 3- to 7-year-old children with LFA, but no correlation was found between the abnormal GMV and their ASD symptoms (27). In addition, one of two earlier studies that did not use VBM methods reported that cerebral GMV was enlarged in both HFA and LFA compared to controls (28). A larger whole-brain volume was also reported in LFA but not in HFA patients who were 1.9- to 5.2-years old (29). Using semi-automated image analyses, it was reported that autistic individuals have a significantly smaller corpus callosum but not cerebellar area (30). The results of these studies were inconsistent, probably because some researchers recruited only adults or only LFA, while others included both children and adults, making comparisons difficult. The second reason is that some of the research used manual or semi-automated imaging analysis programs which can only be applied to specific brain regions. However, the demonstrated differences in brain anatomy may indicate true biological variability in ASD that accurately reflects its clinical heterogeneity. In any case, to our knowledge no previous studies have been carried out using VBM-DARTEL to explore the different patterns of brain anatomy in children with ASD with different IQ levels.

In summary, there are relatively few VBM studies of children with ASD distinguishing HFA from LFA. It remains unknown whether there are differences in brain anatomy associated with the variable clinical phenotypes, and if so, how those variations in brain structure are related to the symptoms of ASD. To address these questions, it will be necessary to recruit relatively large samples of subjects, acquire and analyze the data in a similar manner, and compare the brain anatomy of these subjects across the full spectrum of autism, to reveal any correlations between the brain structure and autistic symptoms that may exist.

One of the aims of our study was to compare the GMV among HFA, LFA, and HC by using the method of VBM-DARTEL; the second aim was to explore the relationship between the clinical phenotype of ASD and autistic symptoms. We hypothesized that LFA has more regions of abnormal brain anatomy than HFA and we assumed that there may be some correlations between specific abnormal brain regions and the severity of autistic symptoms.

## Materials and methods

### Subjects

All subjects were recruited during a 4-year period (2013–2017) from community and clinical sources, including patients from West China Hospital of Sichuan University, special schools, regular schools, and from clinic social skills training groups in Chengdu. Typical developing subjects were recruited from regular schools in Chengdu. After completion of the description of the study purposes to subjects and their parents, written informed consent was obtained. The study was approved by the Ethics Committee of the West China Hospital of Sichuan University. All subjects were ascertained and assessed by a child psychiatrist trained in diagnosis of autism at the Mental Health Center, West China Hospital of Sichuan University. MRI scans, image processing, analysis, and quality control were completed by a professional image technician at the West China Hospital of Sichuan University.

After excluding 16 subjects (including 11 ASD subjects who could not cooperate with MRI scanning even after sedation with chloral hydrate and 5 HC subjects whose MRI images were of low quality) 65 right-handed subjects participated in this study. Among them, there were 19 ASD with HFA (17 males and 2 females, age ranges 5–16 years old, IQ ranges 71–122, excluding 3 Asperger subjects), 19 LFA (15 males and 4 females, age ranges 5–16 years old, IQ ranges 30–67), and 27 HC (26 males and 1 female, age ranges 5–14 years old, IQ ranges 70–130). HFA, LFA, and HC were group matched on gender, age and handedness, and HFA and HC were also matched on IQ.

### Diagnosis

First, the child was diagnosed by an experienced child psychiatrist based on the Fourth Edition of the Diagnostic and Statistical Manual of Mental Disorders (DSM-4). Second, an interview with the child's parents and an assessment of the child was performed by using the Autism Diagnostic Interview-Revised (ADI-R) (31) and Autism Diagnostic Observation Schedule-Generic (ADOS-G) (32).

The ADI-R is an interview about the individual's current social and early childhood and stereotyped, repetitive behaviors and interests and communication development. The ADOS-G includes activities for young children and activities and an interview for older, verbal children, and it is a semi-structured interactive observation session designed to document signs of autism if they are present. Subjects were tested with one of four different modules depending on their age and verbal ability. Because the caretakers of 2 LFA could not come to our hospital and 14 children could not complete the ADOS-G, we failed to collect the results of ADI-R of 2 LFA children and ADOS-G of 14 children (6 LFA and 8 HFA). All patients were medication-naive and met the criteria of DSM-5. ASD subjects were excluded if they had a history of head injury, seizures, birth asphyxia, and metabolic or genetic disorders such as Fragile-X Syndrome.

HC had no history of developmental, learning, neurological, cognitive, or neuropsychiatric problems and they had no history of psychotropic medication use. To confirm that they were typically developing, all of them had extensive testing, including the ADI-R, IQ, and psychiatric testing.

### IQ and handedness

IQ was measured by different versions of intelligence tests for ASD and HC. The Wechsler Preschool Intelligence Scale (WPPSI) was used for the 5- to 6-year-old subjects. The Wechsler Intelligence Scale for Children (WISC-III) was used for 6- to 16-year-old subjects (33). We used the handedness questionnaire as revised by Li Tianxin in 1983. This questionnaire was formulated by asking about the use of hands in daily life of the subjects (34).

### MRI data acquisition and preprocessing

Magnetic resonance images were acquired using a standard quadrature head coil, 8-channel, receive-only on a 3.0 T Verio MRI system (Achieva, Philips, The Netherlands). Foam padding and earplugs were used to diminish the head movement and scanner noise. A number of pulse sequences (T2-weighted, and 2D FLAIR) and image contrasts were collected for clinical review. High-resolution images were obtained with a T1-weighted three-dimensional (3D) spoiled gradient (SPGR) sequence. The parameters were as follows: TR = 8.37 ms; TE = 3.88 ms; flip angle = 7°; in-plane matrix resolution = 256 ^*^ 256; field of view = 24 ^*^ 24 cm2; voxel size = 1 ^*^ 1 ^*^ 1 mm3; thickness = 1 mm; number of slices = 188.

The 15 ASD and 6 HC who could not cooperate with the scanning were sedated using chloral hydrate with parental consent before the MRI was performed. The dosage of chloral hydrate was based on the child's weight (1 ml/kg). The maximum dosage was usually 20 ml. No complications occurred in the subjects who were sedated.

We used MRI Convert software (http://lcni.uoregon.edu/downloads/mri_convert/mriconvert/view) to transform the DICOM format data collected from magnetic resonance scanning. VBM-DARTEL was conducted with SPM8 (http://www.fil.ion.ucl.ac.uk/spm) software on a Matlab (2012Ra) platform. The pre-processing steps were as follows: (1) Examining the images for evidence of anatomical abnormalities in each subject; (2) Setting the image origin to the AC-PC line; (3) Segmentation in the DARTEL procedure (A DARTEL was used to create a customized T1 template of our own images rather than a standard T1 template and the segmented gray matter, GM, and white matter, WM, maps were spatially normalized); (4) Affine transform of segmented brain maps into the MNI space; (5) Modulating the segmented images with the Jacobian determinants derived from the spatial normalization; (6) Using standard smoothing by an 8-mm-full width-half maximum Gaussian kernel. This pre-processing yielded the smoothed modulated normalized data (in the MNI space) used for the statistical analysis

### Statistical analysis

Statistical analysis was performed using the Statistical Package for the Social Sciences (SPSS 22.0 for Windows, IBM Corp., Armonk, NY, USA). Two sample *t*-tests were used to compare the demographic data between the ASD and HC groups. For the sex data, χ2 tests were applied. ANOVA was used to compare the demographic data between the LFA, HFA, and HC groups, and two sample *t*-tests were applied to compare the scores of ADI-R between the LFA and HFA groups. We set the statistical significance level at *F* < 0.05 or *p* < 0.05.

Group differences were evaluated for GMV and white matter volume (WMV) absolute volumes and total cerebral volume (TCV) which were obtained in the brain segmentation step of the VBM-DARTEL pre-processing. TCV was calculated as the sum of the volume of GM, WM and cerebrospinal fluid (CSF).

First, a two sample *t*-test contained in SPM8 was applied to analyze the differences of regional GMV between the ASD group and the HC group. Intelligence quotient, age, sex, and GMV were treated as covariates. VBM analysis was employed using a *p* < 0.001 significance threshold, False Discovery Rate (FDR) uncorrected with an extent threshold of 50 voxels. An absolute threshold mask of 0.2 was used on the GMV. Each individual cluster that showed significant differences between groups was defined as a region of interest (ROI). The GMVs of individual ROIs were extracted from each subject and compared between groups. Pearson's correlation analysis was used to discuss the relationship between the values of GMV and the score of ADI-R and IQ.

Second, the ASD group was divided into a HFA group and a LFA group and ANOVA analysis was used to analyze the differences of regional GMV between the three groups. Age, sex, IQ, and GMV were treated as covariates. Then, *post hoc* analysis was carried out between these three groups. To assess the functional significance of GMV alterations in patients, the values of GMVs in each ROI were correlated with the score of ADI-R, with IQ, age, sex, and GMV being treated as covariates.

## Results

### Demographic characteristics and clinical variables

No significant differences were found between the ASD group and the HC group in demographics, including age (*p* = 0.111), gender (*p* = 0.224); IQ in the ASD group was significantly lower than that in the HC group (*p* < 0.001) (Table 1).

**Table 1:** Demographic characteristics and clinical variables of the ASD and HC groups.

**Characteristics**	**ASD group (*N* = 38)**	**HC group (*N* = 27)**	**F/t**	***p***
Age (years)	9.56 (3.43)	8.33 (2.3)	1,615	0.111^a^
Gender(Male/Female)	32/6	26/1	/	0.224^b^
IQ(WISC-R)	75.84 (25.1)	98.59 (16.64)	−4.107	0.000^a^
ADI-R(M,SD)	42.79 (18.15)	/	/	/
Communication	16.777 (6.47)	/	/	/
verbal interaction	13.86 (5.64)	/	/	/
non-ver interaction	7.44 (4.79)	/	/	/
Restricted repetitive behavior	3.61 (2.23)	/	/	/
TCV(ml)	1448.36 (148.01)	1395.46 (122.77)	1.524	0.132^a^
GMV(ml)	777.51 (68.21)	761.31 (56.69)	1.01	0.316^a^
WMV(ml)	487.39 (70.98)	465.17 (57.83)	1.34	0.185^a^

a *The p-values were obtained by two sample t-tests*.

b *The p-values were obtained by chi-square test*.

No significant differences were found among the LFA, HFA, and HC groups in demographics including age (*p* = 0.16) and gender (*p* = 0.239) except that the IQ of the LFA group was significantly lower than the HC group (*p* < 0.05) and the HFA group (*p* < 0.05). To assess the potential effect of IQ on GM group differences, GM volumes were correlated with IQ within each group. Besides, the score of subscales of ADI-R including communication, verbal interaction, non-verbal interaction, and total score of ADI-R in the LFA group were significantly higher than the HF group (*p* = 0.044, *p* < 0.05, *p* < 0.05, *p* < 0.05) (Table 2).

**Table 2:** Demographic characteristics and clinical variables of the three groups (HFA, LFA, and HC).

**Characteristics**	**LF group (*N* = 19)**	**HF group (*N* = 19)**	**HC group (*N* = 27)**	**F/t**	***p***	***Post hoc***
Age (years)	9.03 (2.99)	10.08 (3.82)	8.22 (2.3)	1.89	0.16^a^	/
Gender (Male/Female)	17/2	4/15	26/1	3.321	0.239^b^	/
IQ(WISC-R)	55.89 (10.83)	95.79 (18.37)	98.59 (16.64)	46.9	0.00^a^	LF<HF,HC
ADI-R(M,SD)	54.94 (14.36)	36.42 (10.38)	/	4.468	0.000^c^	/
Communication	19.05 (7.49)	14.73 (4.71)	/	2.095	0.044^c^	/
Verbal interaction	17.41 (5.36)	10.68 (3.69)	/	4.422	0.000^c^	/
Non-ver interaction	10.52 (4.78)	4.68 (2.71)	/	4.573	0.000^c^	/
Restricted repetitive behavior	3.71 (2.84)	3.52 (1.57)	/	0.238	0.814^c^	/
TCV(ml)	1,460.3 (131.05)	1,436.51 (166.0)	1,395.4 (122.77)	1.288	0.283^a^	/
GMV(ml)	786.96 (58.56)	768.06 (77.11)	761.31 (56.69)	0.926	0.402^a^	/
WMV(ml)	490.51 (61.043)	484.28 (81.32)	465.17 (57.83)	0.927	0.401^a^	/

LF, Low Function; HF, High Function; HC, Healthy Control; TCV, total cerebral volume; GMV, gray matter volume; WMV, white matter volume. Statistical significance level: p < 0.05.

a The p-values were obtained by ANOVA.

b The p-values were obtained by chi-square tests.

c *The p-values were obtained by two-sample t-tests*.

### Quantitative structure MRI

No significant differences were found between the ASD and the HC group with regard to whole TCV (*p* = 0.132), GMV (*p* = 0.316), and WMV (*p* = 0.185) (Table 1).

Also, no significant differences were found among the three groups (HFA, LFA, and HC) with regard to TCV (*p* = 0.283), GMV (*p* = 0.402), and WMV (*p* = 0.401) (Table 2).

### VBM-DARTEL analysis of GMV differences

#### Subjects with ASD vs. controls

Participants with ASD had a large cluster of significant increases in GMV in the left inferior temporal gyrus (*T* = 4.3789, volume = 189) and bilateral middle temporal gyrus (Left: *T* = 4.1618, volume = 283; Right: *T* = 3.8091, volume = 67) as compared to HC. The ASD subjects also had three small areas of decreased GMV including the right precuneus (*T* = −4.6267, volume = 377), right cerebellum anterior lobe (*T* = −3.9329, volume = 84) and the right angular gyrus (*T* = −4.2512, volume = 167) (Table 3).

**Table 3:** Comparison of gray matter volume between the ASD and HC groups.

**Volume (mm^3^)**	**T**	**Talairach coordinates (x,y,z)**	**Anatomical regions**
**ASD**>**HC**			
189	4.3789	−48, 1.5, −42	L Inferior Temporal Gyrus
67	3.8091	36, 9, −42	R Middle Temporal Gyrus BA38
283	4.1618	−57, 3, −21	L Middle Temporal GyrusBA21
**ASD**<**HC**			
84	−3.9329	18, −28.5, −19.5	R Cerebellum Anterior Lobe
377	−4.6267	3, −54, 60	R Precuneus
167	−4.2512	49.5, −64.5, 45	R Angular

*All labels are derived from the Anatomical Automatic Atlas (AAL).The threshold was set at p < 0.001 (uncorrected). x,y,z, coordinates of primary peak locations in the MNI space. L, Left; R, Right; BA, Brodmann Area; LF, Low Function; HF, High Function; HC, Healthy Control*.

#### ANOVA results from subjects with LFA, HFA, and controls

The ANOVA showed six increased GMV clusters among the three groups (significant threshold *p* < 0.001, uncorrected) which included the left inferior temporal gyrus (*T* = 11.4038, volume = 89); left middle temporal gyrus BA21 (*T* = 11.8575, volume = 211); left parahippocampal gyrus BA35 (*T* = 9.5346, volume = 50); right inferior parietal lobule (*T* = 12.3161, volume = 99); right parahippocampal gyrus (*T* = 9.3148, volume = 67); and right precuneus (*T* = 10.8188, volume = 72) (Table 4).

**Table 4:** Comparison of gray matter volume between the LF, HF, and HC groups.

**Volume (mm^3^)**	**T**	**Talairach coordinates (x,y,z)**	**Anatomical regions**
**ANOVA**			
89	11.4038	−48, 3, −42	L Inferior Temporal Gyrus
211	11.8575	−58.5, 4.5, −21	L Middle Temporal Gyrus BA21
50	9.5346	−22.5, −27, −22.5	L Parahippocampa GyrusBA35
99	12.3161	51, −64.5, 48	R Inferior Parietal Lobule
67	9.3148	19.5, −28.5, −21	R Parahippocampal
72	10.8188	3, −54, 60	R Precuneus
**LF**>**HC**			
75	4.5422	−49.5 0 −43.5	L Inferior Temporal Gyrus
210	4.9095	−60 1.5 −12	L Middle Temporal Gyrus BA21
**HF**>**HC**			
87	4.9022	−45 −1.5 −37.5	L Inferior Temporal Gyrus

*All labels are derived from the Anatomical Automatic Atlas (AAL). The threshold was set at p < 0.001 (uncorrected). x,y,z: coordinates of primary peak locations in the MNI space. L, Left; R, Right; BA, Brodmann Area; LF, Low Function; HF, High Function; HC, Healthy Control*.

#### Subjects with LFA vs. controls

**Figure 1:**
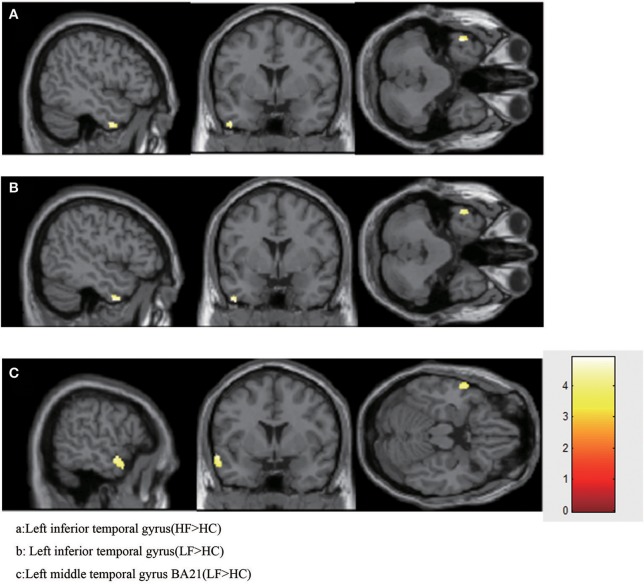
Increased GMV in HFA and LFA group. **(A)** Left inferior temporal gyrus (HF>HC). **(B)** Left inferior temporal gyrus (LF>HC). **(C)** Left middle temporal gyrus BA21 (LF>HC).

Compared with the HC group, increased GMV was found in the LFA group in the left inferior temporal gyrus (*T* = 4.5422, volume = 75); left middle temporal gyrus BA21 (*T* = 4.9095, volume = 210) (Figure 1, Table 4).

#### Subjects with HFA vs. controls

When the HF group was compared with the HC group, increased GMV was found only in left inferior temporal gyrus (*T* = 4.9022, volume = 87) (Figure 1, Table 4).

### Subjects with LFA vs. HFA

No significant increases or decreases of GMV were found when the HF group was compared to the LF group (Figure 1, Table 4).

A two-sample *t*-test was also carried out between every two of the three groups, and the results are shown in Supplemental Table 1.

### Correlation between GMV and clinical symptoms in HFA and LFA patients

**Figure 2:**
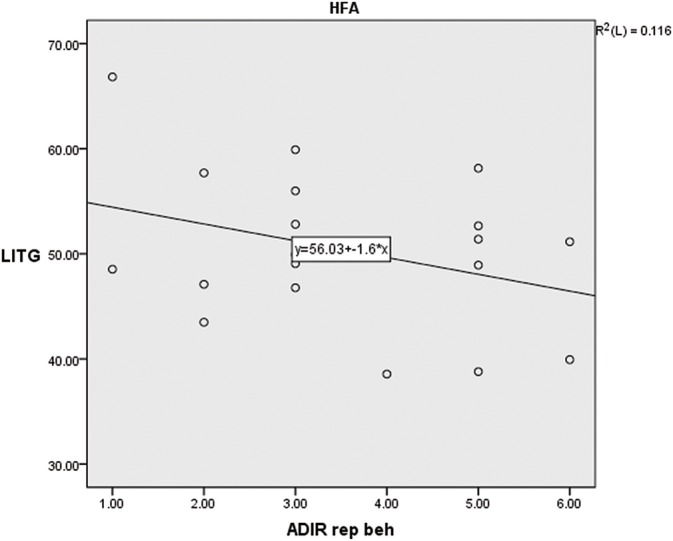
Correlation between LITG and score of repetitive behavoir in HFA group.

A significant negative correlation was found between GMV and the score of repetitive behavior in the HFA group (*p* < 0.01, *r* = −0.649). Yet no significant correlation was found in the LFA or ASD groups (Supplemental Tables 1–3, Figure 2).

### Correlation between GMV and IQ in the ASD and HC groups

No significant correlation was found between GM volumes and IQ and clinical ratings within each group (Supplemental Tables 4–6).

## Discussion

In our study, the unbiased whole brain VBM-DARTEL method was used to assess neuroanatomical differences between children with ASD and HC. We also applied Pearson's correlation analysis to correlate the scores of ADI-R and IQ with abnormalities of brain volume. We found some brain regions that had previously been reported to be abnormal in people with ASD (17, 25, 35). We also found preliminary evidence that some of these anatomical differences occurred in both the LFA and HFA groups whereas others vary according to diagnostic categorization based on IQ. More abnormal brain structures were found in the LFA group, which fitted our previous assumptions that LFA has more abnormal brain anatomy than HFA. In addition, significant correlations between increased GMV and autistic symptoms were found in our study.

We demonstrated increased GMV of the left inferior temporal gyrus (ITG) in both the LFA group and the HFA group. This result remains the same while excluding the influence of IQ on brain structure with IQ as covariates. This suggests that the left ITG is implicated in the pathophysiology of ASD across the autistic spectrum at different IQ levels. In agreement with our study, some studies also found an increase of left temporal lobe (including inferior, middle and superior temporal gyrus) in children with ASD (36–38). But another VBM study found those with autism demonstrated a reduction in superior and inferior temporal gyrus (25). This may be explained by the fact that the subjects in their group were all adults with a mean age of 30, whereas the mean age of our subjects was 9.5 years. It has been reported that infants and children with ASD had a larger brain size than typical of developing children, but that with increased age, the difference in whole brain volume between the two groups was no longer significant (39–41). On the other hand, when the HFA and LFA were combined into one autism group, the heterogeneity of the phenotypes would inevitably be increased. Another study merely found decreased GMV regions including the left ITG, basal forebrain, nucleus accumbens, and cerebellar hemispheres in the LFA group (18). The age of their subjects ranged from 2- to 10-years old, which is younger than the age of our subjects. Moreover, they only included the LFA and HC. As the main brain area to coordinate social communication, emotion and verbal function, the temporal lobe has abnormalities that have been associated with the communication and speech deficits of ASD (42). Early studies showed that the inferior temporal surface plays an important role in orthographic decoding, or was the so-called basal temporal language area (43–45). Later, the concept of the “local combination detector” (LCD) was raised by Dehaene et al. (46), who suggested that the function of the inferior temporal gyrus was related to transmitting large amounts of information back and forth, starting by encoding visual features, then single letters, bigrams, quadrigrams, and finally whole words (46, 47). This was later supported by the ERP study (48). These studies suggest that the ITG may play a key role in learning language at an early age. Left ITG is also supposed to be involved in common object perception. A study found fMRI abnormalities in the inferior temporal and fusiform gyri in face discrimination tasks (49) The abnormal structure of left ITG may explain the difficulty in recognizing faces in ASD patients. More importantly, given this importance of the ITG in language acquisition, our results showing increased GMV of the ITG both in HFA and LFA may partly explain the occurrence of delayed language development before age 3 in both the HFA and LFA groups (25, 28).

In our study, increased GMV of the left middle temporal gyrus (MTG) BA21 was only found in the LFA group, and not in the HFA group. This suggests that, neuroanatomically, the LFA sample may represents a more heterogeneous population than HFA. MTG BA21 is a site involved in auditory processing, language and social perception implicated in ASD-associated behaviors (50). It has been reported that abnormal activity occurs in left middle/superior temporal gyrus, especially in region BA21, which may be the key brain region for speech disorders of ASD children (12). Moreover, the middle temporal cortex and superior temporal gyrus are important sites for perceiving and decoding other people's gaze (26). In postmortem BA21 temporal cortex, a study found that compared to controls, ASD patients exhibited altered protein levels of mitochondria respiratory chain protein complexes, decreased Complex I and IV activities, decreased mitochondrial antioxidant enzyme SOD2, and greater oxidative DNA damage (51). Thus it can be seen that MTG is abnormal in both microstructure and macrostructure, indicating that it may be one of the important causes of ASD. In this study, the subscale scores of ADI-R including communication, verbal interaction and non-verbal interaction in the LFA group were significantly higher than in the HFA group. Therefore, it can be postulated that the more impaired the left temporal gyrus, the more obvious the core symptoms of autism, especially with respect to communication and social interaction deficits. However, we failed to find any correlation between left MTG and core autistic symptoms such as communication deficits. This may be explained by the small sample size.

Finally, in the correlation analysis, we reported significant negative correlations between the GMV of the left ITG and the score of repetitive behavior only in the HFA group. The result remains the same with IQ as covariates. A review summarized the brain regions that related to stereotypical repetitive behaviors reported in previous literature were include basal ganglia, caudate, frontal and temporal cortices. Among these brain areas, basal ganglia was the most commonly reported one and Alterations to frontal and temporal cortices are the most varied and difficult to interpret in relation to RRB, as these regions are also implicated in social and/or communication deficits (52). (53) found faster striatal growth was correlated with more severe repetitive behavior (insistence on sameness) at the preschool age. Rojas et al. (19) reported repetitive behavior was positively associated with the left inferior frontal gyrus, caudate nuclei and right amygdala. In their study, the ASD group included both HFA and LFA subjects and the age ranged from 7.8 to 44. This could introduce variability and reduce the effect sizes for some brain structures. In the present study, we only included children who were 5- to 15-years old, and would have fewer developmental covariant influences that could affect the findings. We did not find the same relationship in the LFA group or in the whole ASD group (HFA combined with LFA). This may be explained by three ways. First, LFA and HFA may be two different subtypes of ASD with different neuroimaging features, so that our results of correlation between GMV of the left ITG and the score of repetitive behavior could only exist in HFA but not in LFA. Second, repetitive behavior reflected by abnormal structures may not particularly evaluated by the ADI-R in LFA patients. Third, the sample size was too small to find any potential relationship in the LFA group.

### Strengths and limitations

The strengths of this study include the enrollment of three groups of young children (LFA, HFA, and HC) and the use of rigorous diagnostic tools, strict and validated imaging methodologies, and exact and original analytic methods. More importantly, so far as we know, this was the first investigation of abnormal brain anatomy in HFA and LFA using VBM, and the first to assess correlations between autistic symptoms, making the results more representative of ASD population. But this study also has limitations. First, all of the subjects were children, so we do not know whether our findings can be generalized to adults. Second, our ASD patients were not followed over time, and so it is difficult to know whether the abnormalities would be altered or extinguished as they grow older. Third, the sample size was relatively small (because we found that it is too difficult for many LFA children to cooperate with MRI scanning). Fourth, we did not include a control group of children with other intellectual disorders for comparison, because the purpose of this research was to find different brain regions in different clinical phenotypes of ASD and HC on various levels of IQ in the same sample. Given the higher proportion of LFA in the overall ASD population, ASD phenotype should include different levels of intelligence to make the results more representative. In attempting to “control” for a domain- non-specific construct such as IQ, variability truly associated with autism could be discarded as “non-specific” (54). In fact, the pathology underlying neurodevelopmental disorders and ASD is itself not well understood (55), so subjects defined on the basis of cognitive function are likely to introduce additional variation related to the causes of the cognitive impairment in ASD research studies (55). Together with the present study, it can be postulated that while understanding biological features of delicate subtypes, research studies on ASD should include subjects with a variety of phenotypes, including different IQ levels, to make the findings more generalizable, so that they can shed light on the biological markers and targeted interventions of ASD in the future.

## Conclusion

Our findings supported the hypothesis that the neurological findings LFA and HFA reflect abnormal function in the same brain regions, but LFA had involvement of more abnormal regions of the brain than HFA. Increased GMV in the left ITG was found in both HFA and LFA, but increased GMV of the left MTG BA21 was found only in the LFA group. Furthermore, there was a significant negative correlation between GMV of the LITG and the score of repetitive behavior in the HFA group. These findings seem to provide a theoretical basis for exploration for biological markers and further targeted interventions in ASD. More importantly, our findings indicate that different levels of IQ should be taken into consideration when discussing the biological markers and targeted intervention of ASD in the future research.

## Author contributions

JC wrote the manuscript of this work. XH, KG, PY, and MS helped recruit and assess the subjects. YH modified the manuscript.

### Conflict of interest statement

The authors declare that the research was conducted in the absence of any commercial or financial relationships that could be construed as a potential conflict of interest.
