# Supplementary material for: Increased Left Inferior Temporal Gyrus Was Found in Both Low Function Autism and High Function Autism
Source: Front Psychiatry. 2018 Oct 30;9:542. doi: 10.3389/fpsyt.2018.00542 (PMC6218606; doi:10.3389/fpsyt.2018.00542)
Supplement: Supplementary file 1 [file Data_Sheet_1.docx]

Supplemental Table 1. Correlation between GMV in different regions and the score of ADI-R (ASD group)

|  | Pre-R | LITG | RMTG | LMTG | Rce | RIPG |
| --- | --- | --- | --- | --- | --- | --- |
| ADIR total | r=0.120 | r=-0.024 | r=-0.190 | r=-0.026 | r=0.050 | r=-0.043 |
| ADIR com | r=-0.064 | r=-0.089 | r=-0.203 | r=0.001 | r=0.071 | r=-0.254 |
| ADIR ver inter | r=0.189 | r=0.087 | r=-0.093 | r=-0.068 | r=0.061 | r=0.169 |
| ADIR nver inter | r=0.183 | r=-0.091 | r=-0.110 | r=-0.129 | r=-0.096 | r=0.009 |
| ADIR rep beh | r=0.055 | r=0.300 | r=0.119 | r=0.270 | r=0.185 | r=0.239 |
| ADIR abno devel | r=0.127 | r=-0.325 | r=-0.322 | r=-0.095 | r=-0.121 | r=-0.189 |

ADI-R com: ADI-R communication; ADI-R ver inter: ADI-R verbal interaction; ADI-R nver inter: ADI-R nonverbal interaction; ADI-R total inter: ADI-R total interaction; ADI-R rep beh: ADI-R repetitive behavior

Pre-R: Right Precuneus; LITG: Left Inferior Temporal Gyrus; RMTG: Right Middle Temporal Gyrus; LMTG: Left Middle Temporal Gyrus; Rce: Right Cerebellum Anterior Lobe; RIPG: Right Inferior Parietal Gyrus

**p* < 0.05 ***p* < 0.01

Supplemental Table 2 Correlation between GMV in different regions and the score of ADI-R (HFA group)

|  | LITG |
| --- | --- |
| ADIR total | r=0.110 |
| ADIR com | r=0.170 |
| ADIR ver inter | r=0.283 |
| ADIR nver inter | r=0.255 |
| ADIR rep beh | r=-0.649** |
| ADIR abno devel | r=-0.347 |

ADI-R com: ADI-R communication; ADI-R ver inter: ADI-R verbal interaction; ADI-R nver inter: ADI-R nonverbal interaction; ADI-R total inter: ADI-R total interaction; ADI-R rep beh: ADI-R repetitive behavior

Pre-R: Right Precuneus; LITG: Left Inferior Temporal Gyrus; RMTG: Right Middle Temporal Gyrus; LMTG: Left Middle Temporal Gyrus; Rce: Right Cerebellum Anterior Lobe; RIPG: Right Inferior Parietal Gyrus

**p* < 0.05 ***p* < 0.01

Supplemental Table 3 Correlation between GMV in different regions and the score of ADI-R (LFA group)

|  | LITG | LMTG |
| --- | --- | --- |
| ADIR total | r=0.002 | r=-0.133 |
| ADIR com | r=-0.493 | r=-0.351 |
| ADIR ver inter | r=0.404 | r=0.125 |
| ADIR nver inter | r=0.401 | r=0.165 |
| ADIR rep beh | r=0.112 | r=-0.104 |
| ADIR abno devel | r=-0.086 | r=0.011 |

ADI-R com: ADI-R communication; ADI-R ver inter: ADI-R verbal interaction; ADI-R nver inter: ADI-R nonverbal interaction; ADI-R total inter: ADI-R total interaction; ADI-R rep beh: ADI-R repetitive behavior

Pre-R :Right Precuneus; LITG: Left Inferior Temporal Gyrus; RMTG: Right Middle Temporal Gyrus; LMTG: Left Middle Temporal Gyrus; Rce: Right Cerebellum Anterior Lobe; RIPG: Right Inferior Parietal Gyrus

**p* < 0.05 ***p* < 0.01

Supplemental Table 4 Correlation between GMV in different regions and the score of IQ (HC group)

|  | Pre-R | LITG | RMTG | LMTG | Rce | RIPG |
| --- | --- | --- | --- | --- | --- | --- |
| VIQ | r=0.420 | r=0.144 | r=0.160 | r=0.305 | r=0.046 | r=0.115 |
| PIQ | r=0.255 | r=0.078 | r=0.443 | r=0.183 | r=0.096 | r=-0.020 |
| FIQ | r=0.380 | r=0.117 | r=0.326 | r=0.265 | r=0.08 | r=0.056 |

Supplement Table 5 Correlation between GMV in different regions and the score of IQ (HFA group)

|  | LITG |
| --- | --- |
| VIQ | r=-0.456 |
| PIQ | r=0.22 |
| FIQ | r=-0.253 |

Supplemental Table 6 Correlation between GMV in different regions and the score of IQ(LFA group)

|  | LITG | LMTG |
| --- | --- | --- |
| VIQ | r=-0.422 | r=-0.263 |
| PIQ | r=-0.401 | r=-0.104 |
| FIQ | r=-0.422 | r=-0.18 |

| LITG: Left Inferior Temporal Gyrus; LMTG BA21: Left Middle Temporal Gyrus BA21; LPG BA35: Left Parahippocampal Gyrus BA35 |
| --- |

**p* < 0.05

***p* < 0.005
